# Do the effects of interventions aimed at the prevention of childhood obesity reduce inequities? A re-analysis of randomized trial data from two Cochrane reviews

**DOI:** 10.1016/j.eclinm.2025.103130

**Published:** 2025-03-04

**Authors:** Jennifer C. Palmer, Annabel L. Davies, Francesca Spiga, Berit L. Heitmann, Russell Jago, Carolyn D. Summerbell, Julian P.T. Higgins, the Inequity in Obesity Prevention Trialists Collaborative Group, Arne Astrup, Valter Cordeiro Barbosa Filho, Mark E. Benden, Lynne Boddy, Laura M. Bogart, Blakely Brown, Angela Carlin, Diana P. Pozuelo Carrascosa, Li Kheng Chai, Clare Drummy, Scott Duncan, Cara Ebbeling, Eva Martos, Stuart Fairclough, Jayne Fulkerson, Douglas A. Gentile, Mary B. Gruber, May Grydeland, Amy S. Ha, Carla Habib Mourad, Kate Gilstad-Hayden, Douglas L. Hill, Gill ten Hoor, Kiya Hurley, Alison Hurst, Nahla Hwalla, Jeannette R. Ickovics, Kate Jolly, Juliana Kain, Susanne Kobel, Viktoria Anna Kovacs, Susi Kriemler, Sarahmarie Kuroko, Alberto Lana, Teresa Shamah Levy, Mairena Sánchez-López, David Lubans, Brian Lynch, Kristine A. Madsen, Claude Marcus, Méndez-Gómez Humarán, Carmen Morales-Ruan, Philip Morgan, Ivan Müller, Robert Newton, Analise Nicholl, Teresia O'Connor, Russell R. Pate, Sebastián Peña, Lorraine B. Robbins, Jardena J. Puder, Thomas Robinson, Rafaela Rosário, Richard Rosenkranz, Jennifer Sacheck, Jo Salmon, Rebecca A. Seguin-Fowler, Nancy E. Sherwood, Hajnalka Takacs, Rachael Taylor, Haixue Wang, Haijun Wang, Robin Whittemore, Simon Wilksch, Zenong Yin, Zhixiong Zhou, Katie Breheny, Deborah M. Caldwell, Sarah Dawson, Yang Gao, Frances Hillier-Brown, Rebecca K. Hodder, Sofus C. Larsen, Theresa HM. Moore, James D. Nobles, Sophie M. Phillips, Jelena Savović, Fanney Thorsteinsdottir, Eve Tomlinson, Luke Wolfenden

**Affiliations:** a Population Health Sciences, Bristol Medical School, University of Bristol, Bristol, UK; b The Research Unit for Dietary Studies, The Parker Institute, Frederiksberg and Bispebjerg Hospital, Copenhagen, Denmark; c Section for General Medicine, Department of Public Health, University of Copenhagen, Denmark; d NIHR Applied Research Collaboration West (ARC West) at University Hospitals Bristol and Weston NHS Foundation Trust, Bristol, UK; e Department of Sport and Exercise Sciences, Durham University, Durham, UK; f Fuse - Centre for Translational Research in Public Health, Newcastle upon Tyne, UK

**Keywords:** Meta-analysis, Health inequities, Childhood obesity prevention

## Abstract

**Background:**

Public health attempts to prevent obesity in children and young people should aim to minimize health inequalities. Two Cochrane reviews examining interventions aiming to prevent childhood obesity found that interventions promoting (only) physical activity have a small beneficial effect on BMI for people aged 5–18 years, as do interventions promoting physical activity alongside healthy eating for 5–11 year olds. We examined whether the effectiveness of the interventions included in these reviews differed according to eight factors associated with inequity: place, race/ethnicity, occupation, gender/sex, religion, education, socio-economic status, and social capital (the PROGRESS framework).

**Methods:**

We collected data on change in BMI (standardized or unstandardized), subgrouped by baseline measures of PROGRESS factors, for intervention and control groups, from trial authors. We calculated the intervention effect per subgroup (mean difference), then contrasted these to estimate interactions between intervention and the baseline factors. We combined interaction estimates for each factor across trials using meta-analyses.

**Findings:**

We collected subgrouped data from 81 trials that took place between 2001 and 2020, involving 84,713 participants. We found no substantial differences in effectiveness of interventions for PROGRESS subgroups in most scenarios. However, in the younger age group (5–11 years), the effect of interventions on standardized BMI appeared to be higher in boys (average difference in mean differences 0.03; 95% CI 0.01 to 0.06; 45 studies, n = 44,740), which was consistent in direction with the BMI effect (average difference in mean differences 0.06 kg/m2; 95% CI −0.02 to 0.13; 31 studies, n = 27,083).

**Interpretation:**

Our findings suggest that those responsible for public health can promote these beneficial interventions without major concerns about increasing inequalities but should be mindful that these interventions may work better in boys aged 5–11 years than girls. More data are needed, so we encourage future trialists to perform subgroup analyses on PROGRESS factors.

**Funding:**

10.13039/501100000272National Institute for Health and Care Research (NIHR).

**Research in context**

**Evidence before this study**

A core principle of any public health guidance is to minimize health inequalities. Two previous studies of the effects of interventions aiming to prevent obesity in children and young people, by promoting physical activity or a healthy diet, found that such interventions do not increase health inequalities. However, these studies used secondary data published in trial reports, limiting the data available for analysis. Two recent Cochrane systematic reviews and meta-analyses of over 200 randomized trials of interventions to prevent obesity in children and young people, found, *on average,* small beneficial effects of physical activity interventions in 5–18 year olds on (standardized and unstandardized) BMI and of combined physical activity and dietary interventions in 5–11 year olds. A previous modelling study found that small beneficial effects such as these, when delivered at scale, have the potential to contribute meaningfully to reducing the prevalence of childhood obesity. However, looking at average effects only may mask differential effects on inequity factors, as represented by the PROGRESS acronym: place, race/ethnicity, occupation, gender/sex, religion, education, socio-economic status, and social capital. Our objective was to collect primary trial results (including results not previously reported) to examine whether effects of interventions vary according to these PROGRESS factors.

**Added value of this study**

To the best of our knowledge, this is the first large-scale meta-analysis to assess the impact of interventions to prevent obesity in children and young people on health inequity using primary data from randomized trials. Data from 81 trials were included, collected directly from the trialists as aggregate data by intervention and by subgroup, and combined in meta-analyses. We found no substantial impact of the interventions on PROGRESS subgroups, although in the younger age group (5–11 years), the effect of interventions (n = 45) on both BMI and standardized BMI was greater in boys.

**Implications of all the available evidence**

Those responsible for public health can be confident in promoting the types of interventions included in this meta-analysis to prevent obesity in children and young people (5–18 years), knowing there is no evidence that these types of interventions increase inequities. One exception was that interventions for younger children may benefit from being as engaging and enjoyable for females as they are for males. We recommend future triallists carry out subgroup analyses of inequity factors to check that interventions are not increasing any health disparities that may exist.

## Introduction

Population levels of overweight and obesity in childhood are a significant global challenge.^1^ From 1990 to 2022, age-standardized prevalence of obesity increased in girls in 186 countries and in boys in 195 countries. In most countries, obesity more than doubled.^2^ Children and adolescents living with obesity are more likely to experience reduced health-related quality of life and, for adolescents, comorbidities including type 2 diabetes mellitus, fatty liver disease and poor mental health.^3^ The primary prevention of childhood obesity is therefore important not only to promote good long term physical and mental health but also to help children realise their full life-time potential.^4^

Inequalities in the prevalence of childhood obesity are widening in the UK and other high-income countries.^5^ There are unfair differences, or inequities, between population subgroups categorized by shared characteristics. The strongest evidence is for socioeconomic status (SES). In high-income countries, higher rates of obesity are present in those with lower SES,^6^^,^^7^ whereas the opposite relationship is observed in most middle-income countries,^7^ and in low-income countries the relationship varies.^8^ Prevalence of childhood obesity is also linked to place of residence,^9^ race and ethnicity^10^ and gender.^11^ There is less evidence for a link with religion or social capital in children, although a connection has been observed in adults.^12^^,^^13^

It is important that attempts to prevent obesity in children recognize these unfair differences. Population-level impacts of interventions can hide differences in effects between subgroups. Even if benefits are seen in all subgroups of the population, interventions will lead to greater inequities if the benefit is greater in the more privileged than the less privileged group.^14^ Interventions are needed that lead to population-level increases in health and wellbeing while also reducing inequities. It is therefore important to understand whether the effectiveness of interventions varies by inequity factors.^15^^,^^16^

Characteristics of intervention content, delivery and implementation have been suggested as reducing or increasing inequities. Targeted (rather than universal) interventions have been proposed as a preferred way to address health disparities,^17^ possibly as a complement to universal interventions.^18^ There is also evidence that upstream interventions, such as those operating within a higher domain of the socio-ecological model^19^ or those on the higher steps of the Nuffield intervention ladder,^20^ are less likely to increase inequities.^21^^,^^22^

A systematic review of SES on obesity-related outcomes found limited evidence from 23 studies for the effect of these interventions on SES inequalities in these outcomes amongst children.^23^ Two recent Cochrane systematic reviews and meta-analyses identified over 200 randomized trials of interventions to prevent obesity in children and young people aged 5–11 and 12–18 years, respectively.^24^^,^^25^ The findings suggested that a range of physical activity interventions, alone or in combination with dietary interventions, can have a modest beneficial effect, on average, on preventing obesity. Cochrane reviews are widely regarded for their quality, with methods that reduce the impact of bias across multiple parts of the review process.^26^^,^^27^ We therefore used the trials identified in these reviews as a basis for exploring health disparities.

This paper describes a re-analysis of this evidence base to examine whether there were differences in the effectiveness of these interventions according to the eight inequity factors identified in the PROGRESS framework: place (of residence), race/ethnicity, occupation (of parents), gender/sex, religion, education (of parents), socio-economic status and social capital.^15^ Our rationale for choosing these factors was that they were derived by an expert group as important sources of inequalities. Furthermore, examination of the published trial reports revealed that many of the PROGRESS inequity factors had been collected at baseline, unlike most of the ‘PROGRESS-plus’ factors (other than age).^28^ However, subgroup analyses based on these baseline characteristics were seldom reported. It was therefore unclear whether obesity prevention interventions affect the size of the inequity gap, either positively or negatively. To address this uncertainty, we collected subgroup data from as many trials as possible and used meta-analysis to examine differences with higher statistical power than would be possible in any individual trial. We focus on body mass index (BMI, both standardized by age and sex and unstandardized) as this is the most widely measured outcome in obesity prevention trials.

## Methods

### Selection of included trials

We identified trials through the two recent Cochrane systematic reviews on interventions for preventing obesity in children aged 5–18 years.^24^^,^^25^ In brief, these reviews sought studies that: (i) were individually- or cluster-randomized trials; (ii) recruited children with a mean age between 5 and 18; (iii) measured BMI or standardized BMI z-score (zBMI) assessed at baseline and at least 12 weeks after baseline; (iv) examined an intervention whose main aim was to change at least one from: diet, physical activity, sedentary behaviour, sleep, play or structured exercise to help prevent childhood obesity; and (v) published primary results in 1990 or later. The included trials took place between August 2001 and April 2020. We sought only comparisons of active interventions against a control group. Our protocol for the project was published before we began collecting data.^29^

#### Registration

This is part of a wider study. The overall protocol can be accessed here: https://fundingawards.nihr.ac.uk/award/NIHR131572.

### Data collection

We extracted subgroup outcome data from publications where they were reported. Where relevant subgroup data had not been reported, the corresponding authors of the trials were emailed to request these data. The emails were tailored to each recipient to include details of the main published report for the trial in question and any information we had already extracted from the trial reports about the impact of baseline PROGRESS factors. The email included a link to the protocol for our investigation^29^ and access to a data collection table into which outcome data suitable for our analyses could be entered. If we received automatic replies from addresses no longer valid, we took steps to locate the corresponding author online. Where corresponding authors could not be located, we tried to locate other co-authors online. All emails were followed up with reminders.

We sought data subgrouped by baseline measures of PROGRESS factors. For each PROGRESS factor, trialists were asked to divide the trial participants into exactly two subgroups as described in Table 1. Since our primary interest was in the direction of differences between subgroups, the precise cut off for dichotomization was not critical. To maximize statistical power of the analyses, we preferred a dichotomization that resulted in roughly a 50:50 split of participants. Our preference was for inequity factors to be measured and dichotomized at the individual child level, but we accepted group-level categorizations for each child (e.g., at school-level) if that is how the factor was measured. Data were also requested about baseline weight status and we will report the results of our investigation into baseline weight status elsewhere.

**Table 1:** Dichotomization of baseline PROGRESS factors into subgroups.

Factor	Categories	Comments
Place of residence	• Urban; versus • Rural/coastal	As most trials were school based, we expected a high level of homogeneity among participants within each trial, particularly for secondary school-based trials, preventing a meaningful investigation of this factor.
Race/ethnicity/culture/language	• Dominant (most privileged or dominant race/ethnic/culture/language group in the population under study); versus • Minority (other race/ethnic/culture/language group)	We asked trialists to select a demographic characteristic such as race, ethnicity or culture that best defines the group of people who are considered to be the most privileged or dominant within the wider population setting of the trial (for example, White British in the UK). We requested that participants were subgrouped based on whether they did or did not fall into that category.
Occupation (parental)	Any dichotomous split, e.g., • Higher (professional or managerial occupation); versus • Lower (other occupation)	We re-interpreted the PROGRESS factor ‘occupation’ to refer to occupation of the parent(s) rather than the child. We asked trialists to choose an appropriate dichotomization of parental occupation, for example, comparison groups 1–3 of the International Standard Classification of Occupations against other groups.
Gender/Sex	• Male; versus • Female	
Religion	• Dominant (state religion or less oppressed religion in the population under study); versus • Minority (other religion)	Where appropriate this could refer to more than one religion, for example in the UK the first group might comprise ‘Christian’ and ‘Non-religious’. The categorization most likely refers to the child’s parents’ religion.
Education (parental)	Any dichotomous split, e.g., • Higher (higher education); versus • Lower (no higher education)	We re-interpreted the PROGRESS factor ‘education’ to refer to education level of the parent(s) rather than the child. We asked trialists to choose an appropriate dichotomization of parental education.
Socioeconomic status	• Higher; versus • Lower	We asked trialists to choose an appropriate measure (continuous or ordinal) of socio-economic status based on their trial population and the data collected. We asked them to select a dichotomization that gave approximately equal numbers of participants in each subgroup, for example splitting the population at the median.
Social capital	Any dichotomous split, e.g., • Higher (two parents living in the same residence as the child); versus • Not two parents living in the same residence as the child.	Social capital refers to support available through informal social networks. In young people, this is largely related to family structure and the form and quality of family relationships.^28^ We gave as an example the split between two parents living in the same residence as the child versus no parents or separated from parents (e.g., children in care or living with other family members), single parent or separated parents living in different locations.

For each subgroup, raw means and standard deviations (SDs) were collected from each intervention arm of each trial (unadjusted for clustering or other variables). In order of preference, the data collection form asked for (i) mean change from baseline, (ii) baseline and follow-up means with the corresponding correlation coefficient (where available), or (iii) follow-up means. It also requested information about clustering (cluster sizes and intraclass correlation coefficients (ICCs)) so that adjustments for clustering could be made in the analyses. Trialists were asked to provide both zBMI and BMI data where available; zBMI was our preferred outcome for the analysis although many trials had collected only BMI. For trials that measured outcomes at multiple follow-up times, data were requested only for the follow-up time closest to 12 months, which was near the middle of the distribution of follow-up times.

We additionally coded individual trials for characteristics shown to be associated with reducing or increasing inequities: whether the intervention was targeted or universal; the domain of the socio-ecological model it addresses^19^; the step on the Nuffield intervention ladder it addresses^20^; whether it included an explicit component aiming to change the structural environment of the child; and the degree of public engagement and involvement in its development.

Our quality control measures to verify data included (i) checking that changes in zBMI/BMI between baseline and follow-up means and SDs were within the expected range (and we asked trialists to double check if not) and (ii) sharing a draft manuscript with trialists to review their results prior to submission.

### Assessment of risk of bias

The Cochrane review had used the RoB 2 tool^30^ to assess risk of bias in each result. Because the biases addressed by RoB 2, as well as publication bias, may be expected to apply similarly to each subgroup, we would expect many of them to cancel out in our comparisons of subgroups. We therefore supplemented the RoB 2 results with two additional assessments focussing on potential biases in the comparison of two subgroups (i) completeness of data including the extent to which sought results were available (i.e., bias due to missing subgroup data that were extracted from the main analysis papers or provided by trialists) and (ii) classification of participants into subgroups according to thresholds determined by trialists (bias in selection of the subgroup analysis result). Risk-of-bias assessments were undertaken by researchers at the University of Bristol who were not involved in any of the trials.

### Data analysis

Our main analyses were two-stage meta-analyses performed using R. For each baseline factor of interest and each trial, we first calculated the intervention effect per subgroup. These were obtained as mean differences (MD) in zBMI (or BMI) where, depending on the data available, the mean from each intervention group was (i) change from baseline provided by the trialists, (ii) change from baseline calculated from baseline and follow-up means and a correlation coefficient provided by the trialists, (iii) change from baseline calculated from baseline and follow-up means and an imputed correlation coefficient, (iv) follow-up means. Based on observed correlations between baseline and follow-up in trials in our original dataset,^24^^,^^25^ we imputed a value of 0.9 for scenario (iii). We then calculated the difference in intervention effects between the two subgroups. This estimates the interaction between the intervention and the factor defining the subgroup. Since the subgroups are independent, an estimate of the variance of the interaction is given by the sum of variances of the subgroup-specific effect estimates.

Next, we combined the interaction estimates across trials using standard meta-analysis procedures.^31^ We performed a random-effects meta-analysis to allow for heterogeneity in the estimated interaction parameters. The null hypothesis for each meta-analysis was that the subgroup covariate has no impact on the intervention effect (i.e., the interaction parameter is zero). Using the summary estimate and its standard error from the meta-analysis, we performed a simple Z test of this null hypothesis. The point estimate from the meta-analysis quantifies the average extent to which the intervention effect is impacted by the covariate. Compared with the test of the null hypothesis, practical interpretation of this result requires stronger assumptions about the similarity of relationships across trials. We used the I^2^ statistic to measure the extent to which results were consistent across trials and the P value (P_het_) from a chi-squared test to examine strength of evidence of heterogeneity in the interactions across trials.^32^ Evidence of heterogeneity indicates that the impact of the factor is importantly different in different contexts.

For cluster-randomized trials, we adjusted the standard error of the mean differences to account for clustering using methods described previously.^33^ Where available, this adjustment made use of the ICCs reported by the trialists. Where these were not provided, we used an imputed value of 0.02 based on ICCs reported in other trials, and we performed sensitivity analyses with ICCs of 0 and 0.04. For multi-arm trials, we combined intervention groups following the methods described in the Cochrane Handbook.^34^

We performed a Supplementary analysis undertaking meta-analyses separately for the subsets of trials from high-income countries and low- or middle-income countries (according to the World Bank classification), because the association of some PROGRESS factors with obesity may differ between settings (for example, lower socioeconomic status is associated with more obesity in high income countries but often with less obesity in other countries^7^). We additionally performed subset analyses (that were not specified in the protocol) for gender/sex according to whether the intervention targeted diet, physical activity (physical activity, sedentary behaviour or sleep, play or structured exercise) or a combination of diet and physical activity.

### Role of funding

This work was funded by the National Institute for Health and Care Research (NIHR) Public Health Programme [grant number: NIHR131572]. JPTH was supported in part by his Senior Investigator Award from the NIHR [grant number: NIHR203807]. The views expressed in this paper are those of the authors and do not necessarily reflect those of the NIHR or Department of Health and Social Care. The funder had no role in the study design, analyses, interpretation, writing of the report or decision to submit the article for publication.

## Results

### Response rates and included trials

**Fig. 1:**
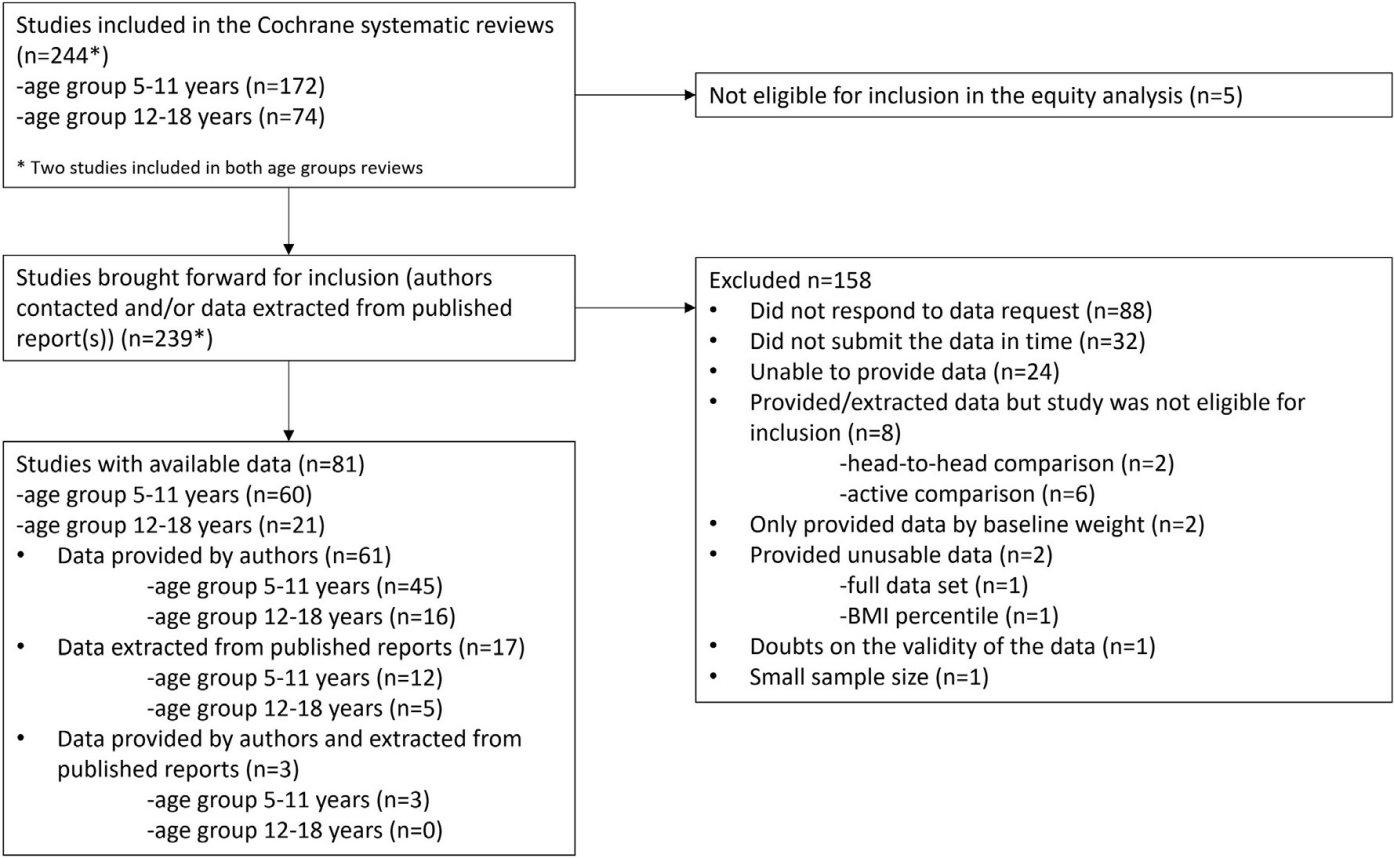
Trial selection process.

We summarize the trial selection process in Fig. 1. From the 244 trials included in the two Cochrane reviews, we excluded five that performed only head-to-head comparisons without a control group, leaving 239 eligible trials. From our attempts to obtain subgrouped outcome data from the trialists, a response was received from the corresponding (or senior) author of 138 trials (58% response rate for contact). We obtained subgrouped outcome data eligible for inclusion in our analysis from the authors of 64 trials (27% response rate for data collection). We were able to extract subgrouped outcome data from 20 publications, including three trials for which authors provided additional data. We therefore included 81 trials (34% of eligible) in the analyses presented below.

### Characteristics of included trials

Brief characteristics of the 81 included trials involving 84,713 participants, including information about the types of interventions used, are provided in Supplementary Table S1. Sixty trials (74%) were conducted in children aged 5–11 years and 21 in children and adolescents aged 12–18 years. Sixty-five trials (80%) randomized clusters of children and 16 (20%) randomized individuals. Sixty-seven (83%) were conducted in high-income and 14 (17%) in middle-income countries (11 in upper-middle-income countries and 3 in lower-middle-income countries). Trial locations were the United States for 23 trials (28%), Australia for 15 (19%), China for seven (9%), United Kingdom for six (7%) and the rest of Europe for 15 (19%). The remining 15 locations included one in Egypt and two in Lebanon. In 39 trials (48%), the interventions aimed to change both dietary and activity behaviours, in 30 (37%) physical activity behaviours only and in ten (12%) dietary behaviours only; two trials (3%) had multiple intervention arms and implemented different types of interventions. Most of the trials (62, 77%) implemented the intervention entirely or mainly at school (including after school programs) and six (7%) were implemented entirely or mainly at home. Three (4%) were implemented within a clinical setting and the remaining 10 (12%) in the community or other setting. Three interventions included telehealth.

The coding of individual trials for characteristics associated with increasing or decreasing inequities is provided in Supplementary Table S2. Most interventions, in total 60 (74%), were universal and 21 (26%) were targeted; some universal interventions were conducted in relatively low-income areas. Nearly all interventions (74/81) operated in the organization or community domains of the socio-ecological model; none in the society or public policy domains and seven in the interpersonal domain. Most interventions (76/81) guided or enabled behaviour change; four included significant restriction (relating to types of foods and beverages available at school) and one provided information via a brief counselling session on healthy weight (at a dental check-up). Around half of the interventions (39/81) involved a change in the school structural environment (for example, changing the catering or school shop layout). Most (56/81) did not report any evidence of public involvement and engagement; 15 reported some degree of consultation and a further ten reported some degree of consultation that included consultation with children. None of the interventions reported using co-production or were user controlled.

Availability of data from individual trials for each PROGRESS characteristic is summarized in Table 2. The mean follow-up time for these outcomes was 10 months (SD 5 months), with the shortest follow-up time being 3 months and the longest 24 months.

**Table 2:** Outcome data obtained from each trial.

Trial ID	Age group	Place of residence	Race/ethnicity/culture/language	Occupation	Gender/sex	Religion	Education	Socioeconomic status	Social capital	Sample size (at randomization)	Follow-up (months)
Adab 2018	5–11	zBMI, BMI	*zBMI*^a^	zBMI, BMI	*zBMI*^a^	zBMI, BMI	zBMI, BMI	*zBMI*^a^	zBMI, BMI	2462	15
Barbosa Filho 2017	12–18	BMI	.	.	.	.	BMI	BMI	.	1272	4
Barnes 2015	5–11	.	.	.	.	.	.	zBMI	.	48	5
Bogart 2016	12–18	.	zBMI, BMI	.	zBMI, BMI	.	.	zBMI, BMI	.	4022	24
Breheny 2020	5–11	.	*zBMI*^a^	.	*zBMI*^a^	.	.	*zBMI*^a^	.	2280	12
Brown 2013	5–11	.	.	.	zBMI, BMI	.	.	.	.	76	3
Chai 2019	5–11	BMI	.	BMI	BMI	.	BMI	BMI	.	46	6
Damsgaard 2014	5–11	.	zBMI, BMI	.	zBMI, BMI	.	.	zBMI, BMI	.	823	3
Dewar 2013	12–18	.	BMI	.	.	.	.	.	.	357	12
Drummy 2016	5–11	.	.	.	BMI	.	.	.	.	107	3
Duncan 2019	5–11	.	.	.	BMI	.	.	.	.	1200	6
Ebbeling 2006	12–18	.	BMI	.	BMI	.	.	BMI	.	103	6
El Ansari 2010	12–18	.	.	.	*BMI*	.	.	.	.	160	3
Fairclough 2013	5–11	.	.	.	zBMI, BMI	.	.	zBMI, BMI	.	318	7
Farmer 2017	5–11	.	zBMI	.	zBMI	.	.	zBMI	.	902	12
Fulkerson 2015	5–11	.	zBMI	.	zBMI	.	.	zBMI	.	160	12
Fulkerson 2022	5–11	.	zBMI	.	zBMI	.	zBMI	zBMI	.	114	9
Gentile 2009	5–11	.	BMI	.	BMI	.	.	BMI	.	1323	6
Griffin 2019	5–11	.	zBMI, BMI	.	zBMI, BMI	zBMI, BMI	zBMI, BMI	zBMI, BMI	.	61	6
Grydeland 2014	5–11	.	zBMI, BMI	.	zBMI, BMI	.	zBMI, BMI	zBMI, BMI	.	2165	20
Ha 2021	5–11	.	.	.	zBMI, BMI	.	zBMI, BMI	zBMI, BMI	.	160	10
Habib-Mourad 2014	5–11	.	.	.	*BMI*^a^	.	.	.	.	374	3
Habib-Mourad 2020	5–11	.	.	.	zBMI	.	.	zBMI	.	1239	19
Haerens 2006	12–18	.	.	.	*zBMI*^a^*, BMI*^a^	.	.	.	.	2840	12
Hollis 2016	12–18	.	.	.	*zBMI*^a^*, BMI*^a^	.	.	.	.	1233	12
Hopper 2005	5–11	.	zBMI	.	zBMI	.	.	.	.	238	8
Ickovics 2019	5–11	.	zBMI	.	zBMI	.	.	zBMI	.	756	12
Jones 2015	5–11	.	.	.	*zBMI*^a^*, BMI*^a^	.	.	.	.	37	12
Kain 2014	5–11	.	.	.	zBMI	.	.	.	.	651	14
Kennedy 2018	12–18	.	BMI	.	BMI	.	.	BMI	.	607	12
Kobel 2017	5–11	.	zBMI	.	zBMI	.	zBMI	zBMI	.	525	12
Kriemler 2010	5–11	zBMI, BMI	.	.	zBMI, BMI	.	zBMI, BMI	.	.	502	10
Kuroko 2020	12–18	.	zBMI	.	zBMI	.	.	zBMI	.	164	12
Lana 2014	12–18	.	BMI	.	BMI	.	.	.	.	2001	5.5
Levy 2012	5–11	.	.	.	zBMI	.	.	zBMI	.	1020	6
Li 2010	5–11	.	.	.	*zBMI*^a^*, BMI*^a^	.	.	.	.	4700	12
Li 2019	5–11	.	.	.	*zBMI*^a^	.	.	.	.	1641	12
Liu 2019	5–11	.	.	.	zBMI, BMI	.	.	.	.	1889	12
Liu 2022	5–11	.	.	.	zBMI, BMI	.	.	zBMI, BMI	.	1392	9
Lloyd 2018	5–11	.	.	.	zBMI, BMI	.	.	zBMI, BMI	.	1324	18
Lubans 2021	12–18	.	zBMI	.	zBMI	.	.	zBMI	.	670	12
Lynch 2016	5–11	.	BMI	.	BMI	.	.	BMI	.	51	4
Madsen 2013	5–11	.	.	.	zBMI, BMI	.	.	zBMI, BMI	.	156	8
Marcus 2009	5–11	.	.	.	zBMI	.	.	.	.	3135	20
Martinez-Vizcaino 2014	5–11	.	BMI	.	*BMI*^a^	.	.	.	.	1592	9
Martinez-Vizcaino 2020	5–11	.	BMI	.	*zBMI*^a^*, BMI*^a^	.	.	BMI	.	2407	8
Morgan 2011	5–11	.	.	.	zBMI	.	.	.	.	71	6
Morgan 2014	5–11	.	.	.	zBMI	.	.	zBMI	.	132	3
Morgan 2019	5–11	.	.	.	.	.	.	zBMI	.	153	9
Muller 2019	5–11	.	.	.	zBMI, BMI	.	.	zBMI, BMI	.	1009	15
Nemet 2011b	5–11	.	.	.	*BMI*^a^	.	.	.	.	342	12
Newton 2014	5–11	.	zBMI	.	zBMI	.	.	zBMI	.	27	3
Nicholl 2021	5–11	.	.	.	zBMI, BMI	.	.	zBMI, BMI	.	49	3
O'Connor 2020	5–11	.	.	.	zBMI, BMI	.	.	zBMI, BMI	.	64	3
Pate 2005	12–18	.	BMI	.	.	.	.	.	.	2744	12
Pena 2021	5–11	.	zBMI	.	zBMI	.	.	zBMI	.	2022	7
Pfeiffer 2019	12–18	.	BMI	.	.	.	.	BMI	.	1519	4
Puder 2011	5–11	.	.	.	BMI	.	.	BMI	.	652	10
Rosario 2012	5–11	.	.	.	BMI	.	.	.	.	464	12
Rosenkranz 2010	5–11	.	zBMI	.	.	.	.	zBMI	.	76	6
Rush 2012	5–11	.	*zBMI*^a^	.	*zBMI*^a^	.	.	.	.	6456	24
Sacchetti 2013	5–11	.	.	.	*BMI*^a^	.	.	.	.	497	24
Salmon 2022	5–11	.	zBMI	.	zBMI	.	.	zBMI	.	593	18
Seguin-Fowler 2021	5–11	.	zBMI	.	zBMI	.	.	.	.	305	5
Sekhavat 2014	5–11	.	.	.	*zBMI*^a^*, BMI*^a^	.	.	.	.	168	9
Sherwood 2019	5–11	.	zBMI	.	zBMI	.	.	zBMI	.	421	12
Shomaker 2019	12–18	.	BMI	.	BMI	.	.	BMI	.	54	6
Singh 2009	12–18	.	.	.	*BMI*^a^	.	.	.	.	1108	12
Smith 2014	12–18	.	BMI	.	.	.	.	.	.	361	9
Stettler 2015	5–11	.	zBMI	.	zBMI	.	.	.	.	173	12
Takacs 2020	12–18	.	.	.	BMI	.	.	.	.	229	12
Tanskey 2017	5–11	.	zBMI	.	zBMI	.	.	zBMI	.	769	11
TenHoor 2018	12–18	.	.	.	zBMI	.	.	.	.	695	12
Vizcaino 2008	5–11	.	.	.	*BMI*^a^	.	.	.	.	1409	9
Weeks 2012	12–18	.	.	.	*BMI*^a^	.	.	.	.	99	8
Wendel 2016	5–11	.	zBMI	.	zBMI	.	.	.	.	173	12
Wilksch 2015	12–18	.	.	.	BMI	.	.	.	.	1441	12
Williamson 2012	5–11	.	.	.	*zBMI*^a^	.	.	.	.	1473	18
Xu 2017	5–11	.	.	.	*zBMI*^a^*, BMI*^a^	.	.	.	.	7717	12
Yin 2012	5–11	.	BMI	.	BMI	.	.	BMI	.	1187	8
Zhou 2019	12–18	.	.	.	BMI	.	.	BMI	.	758	8
Total: zBMI	5–11	2	21	1	45	2	7	29	1		
	12–18	0	3	0	5	0	0	3	0		
	All	2	24	1	50	2	7	32	1		
Total: BMI	5–11	3	8	2	32	2	6	17	1		
	12–18	1	9	0	13	0	1	7	0		
	All	4	17	2	45	2	7	24	1		

BMI = body mass index; zBMI: age- and sex-standardized body mass index.

a Extracted from the published articles.

### Risk of bias

Risk of bias in main trial results (as reported in the Cochrane reviews) was ‘Low’ for seven trials (9%), ‘Some concerns’ for 46 (57%), and ‘High’ for 28 (35%). Assessment of the completeness of data (i.e., bias due to missing subgroup data that were extracted from the main analysis papers or were provided by trialists) resulted in ‘Low’ risk of bias for 69 trials (85%), and ‘Some concerns’ for five trials (6%). Of the remaining trials, six trials (7%) were judged differentially for different subgroups results: four trials were ‘Low’ or ‘Some concerns’, and two trials were ‘Low’ or ‘High’. We were not able to assess one trial for completeness of data as the sample size of the overall results were not reported in the main article. Assessment of risk of bias arising from classification of participants into subgroups according to thresholds determined by trialists (bias in selection of the subgroup result) resulted in all trials being judged at ‘Low’ risk of bias.

### Meta-analyses

**Fig. 2:**
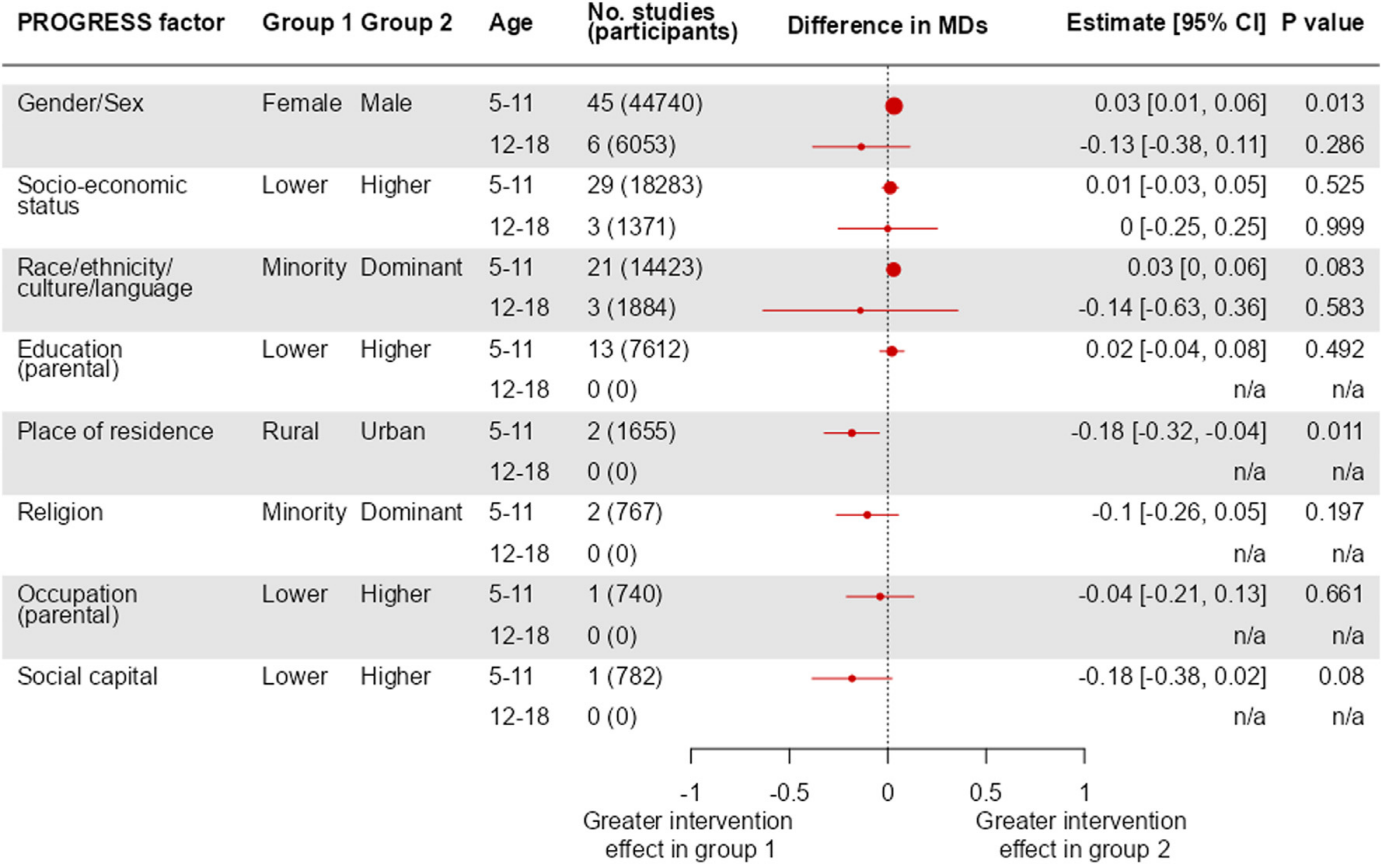
Summary of results for age- and sex-standardized BMI (zBMI): estimates of interaction, expressed as difference in intervention effect (itself expressed as a mean difference) between two inequity factor-based subgroups.

**Fig. 3:**
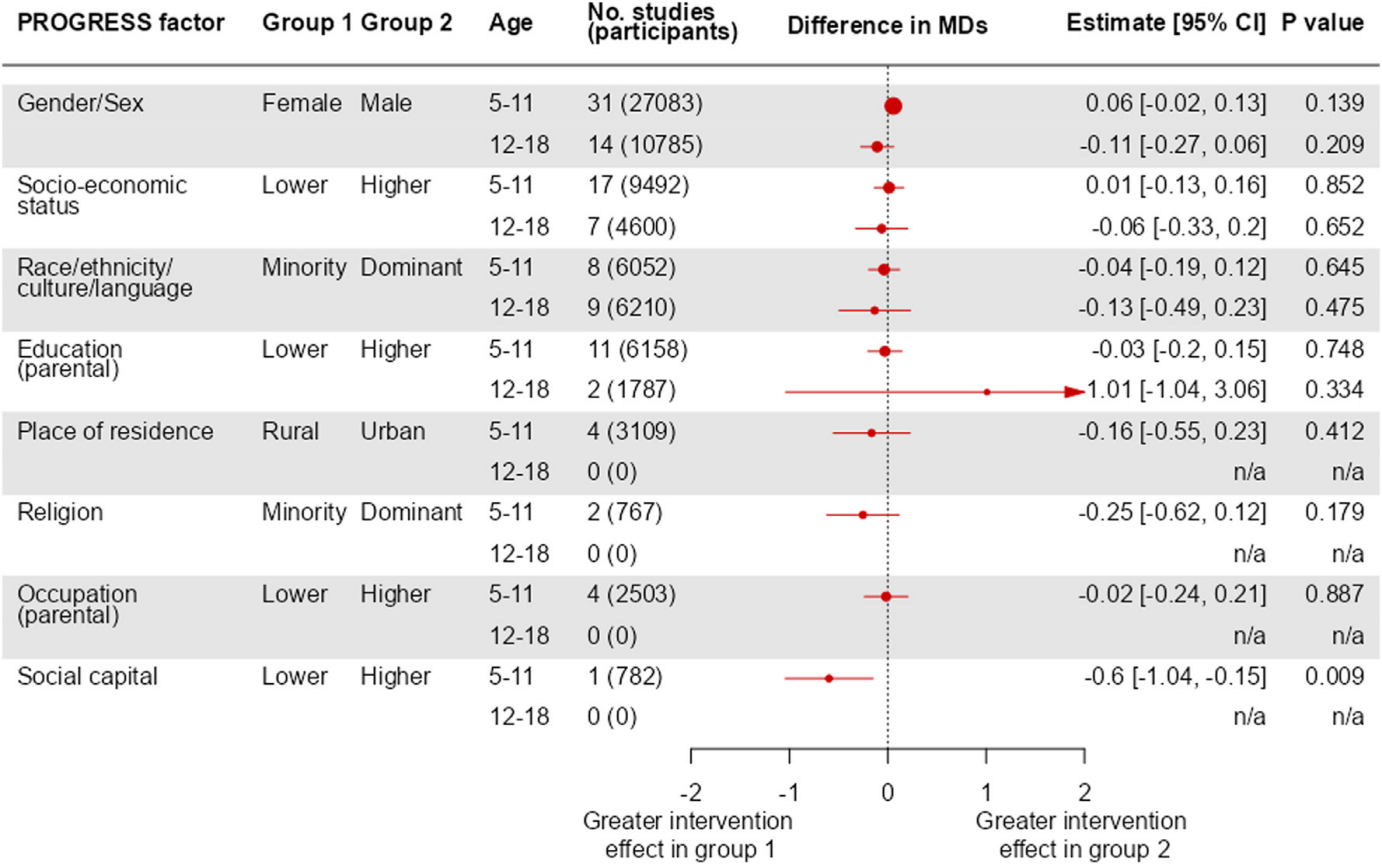
Summary of results for BMI: estimates of interaction, expressed as difference in intervention effect (itself expressed as a mean difference) between two inequity factor-based subgroups.

Forest plots summarizing the estimated average magnitude of the difference in mean differences, and 95% confidence intervals around this estimate, for the eight PROGRESS factors are provided in Fig. 2 for zBMI and Fig. 3 for BMI. We present results according to the total number of trials providing data for analyses. To provide context for interpreting the magnitudes of effect, we note that across all included participants (from all studies and arms) the SD of zBMI was 1 and the SD of BMI was 3.52, with both factors following approximate normal distributions. Since differences up to a fifth of a SD are generally regarded as small,^35^ the effect magnitude might be interpreted as ‘small’ if the zBMI difference is shifted by less than 0.2 or the BMI difference by less than 0.7. Sensitivity analyses using different ICCs in the adjustment for clustering yielded no meaningful differences; confidence intervals were, as is to be expected, slightly narrower for ICC = 0 and slightly wider for ICC = 0.4.

#### Gender/sex

In the younger age group (5–11 years), we included 45 trials (44,740 participants) with zBMI data and 31 trials (27,083 participants) with BMI data, subgrouped by gender/sex. The test for interaction provided evidence of a differential effect of interventions between males and females on zBMI (P = 0.01, Fig. 2), although not on BMI (P = 0.1, Fig. 3). In both analyses the intervention effect was greater in males than females. Results from individual trials are reported in Supplementary Figs. S1 and S2 for zBMI and BMI, respectively. Results from subset analyses provided no evidence that the impact of gender/sex differed for different types of interventions (diet, activity or diet and activity) (test for subset differences P = 0.4 for zBMI and P = 0.5 for BMI) or for different income country statuses (P = 0.8 for zBMI and P = 0.9 for BMI) (Supplementary Table S4).

In the older age group (12–18 years), we included six trials (6053 participants) with zBMI data and 14 trials (10,758 participants) with BMI data. The test for interaction provided little evidence of a differential effect of interventions on zBMI (P = 0.3, Fig. 2) and BMI (P = 0.2, Fig. 3), between males and females. Results from individual trials are reported in Supplementary Figs. S3 and S4 for zBMI and BMI, respectively. Results from subset analyses provided some evidence that the impact of gender/sex differed for different type of interventions for zBMI as an outcome but not on BMI (test for subset differences P = 0.001 for zBMI, P = 0.8 for BMI), with interventions targeting both diet and activity being more effective in males. There was little evidence that the impact of gender/sex differed by country income status (P = 0.4 for BMI). We were not able to perform a subset analysis for zBMI because all trials in the older age group that provided zBMI data by sex were from high income countries (Supplementary Table S5).

#### Socioeconomic status

In the younger age group (5–11 years), we included 29 trials (18,283 participants) with zBMI data and 17 trials (9492 participants) with BMI data subgrouped by socioeconomic status. There was no evidence of a differential effect of interventions between children with higher and lower socioeconomic status (P = 0.5 for zBMI, Fig. 2; P = 0.9 for BMI, Fig. 3). Results from individual trials are reported in Supplementary Figs. S5 and S6 for zBMI and BMI respectively. Results from subset analyses provided no evidence that the impact of socioeconomic status differed for different income country status (P = 0.9 for zBMI and P = 0.2 for BMI, Supplementary Table S4).

In the older age group (12–18 years), we included 3 trials (1308 participants) with zBMI and 7 trials (4600 participants) with BMI data. Again, the test for interaction provided no evidence of a differential effect of interventions between participants with higher socioeconomic status and participants with lower socioeconomic status (P = 1 for zBMI, Fig. 2; P = 0.7 for BMI, Fig. 3). Results from individual trials are reported in Supplementary Figs. S7 and S8 for zBMI and BMI respectively. Results from subset analyses provided little evidence that the impact of socioeconomic status differed for different income country status (P = 0.8 for BMI, Supplementary Table S5). We were not able to perform a subset analysis for zBMI because all trials in the older age group that provided zBMI data by socioeconomic status were from high income countries.

#### Race/ethnicity/culture/language

In the younger age group (5–11 years), we included 21 trials (14,423 participants) with zBMI and eight trials (6052 participants) with BMI data, subgrouped by race/ethnicity/culture/language. The test for interaction provided little evidence of a differential effect of interventions on zBMI (P = 0.08, Fig. 2) and BMI (P = 0.6, Fig. 3), between participants in the dominant ethnic group and participants in the minority ethnic group. Results from individual trials are reported in Supplementary Figs. S9 and S10 for zBMI and BMI, respectively.

In the older age group (12–18 years) we included three trials (1884 participants) with zBMI and nine trials (6210 participants) with BMI data. The test for interaction provided no evidence of a differential effect of interventions on zBMI (P = 0.6, Fig. 2) and BMI (P = 0.5, Fig. 3), between participants in the dominant ethnic group and participants in the minority ethnic group. Results from individual trials are reported in Supplementary Figs. S11 and S12 for zBMI and BMI, respectively.

#### Education (parental)

In the younger age group (5–11 years), 13 trials (7216 participants) with zBMI and 11 trials (6158 participants) with BMI provided data subgrouped by parental education status. The test for interaction did not indicate a differential effect of interventions on zBMI (P = 0.5, Fig. 2) or BMI (P = 0.7, Fig. 3), between participants in the higher parental education group and participants in the lower parental education group. Results from individual trials are reported in Supplementary Figs. S13 and S14 for zBMI and BMI, respectively. Results from subset analyses did not provide evidence that impact of parental education differed for different income country status (P = 0.3 for zBMI and P = 0.1 for BMI, Supplementary Table S4).

In the older age group (12–18 years), two trials (1718 participants) provided BMI data and no trials zBMI data. The test for interaction showed no evidence of a differential effect of interventions on BMI (P = 0.3, Fig. 3), between participants in the higher parental education group and participants in the lower parental education group. Results from individual trials are reported in Supplementary Fig. S15. Results of subset analyses support little evidence that the impact of parental education differed for different income country status (P = 0.1, Supplementary Table S5).

#### Place of residence

In the younger age group (5–11 years), we included two trials (1655 participants) with zBMI and four trials (3109 participants) with BMI data, subgrouped by place of residence (urban versus rural). The test for interaction provides some evidence of a differential effect of interventions between participants living in urban locations and participants living in rural locations on zBMI (P = 0.01, Fig. 2) but not BMI (P = 0.4, Fig. 3), with the intervention appearing to be more effective in participants living in rural locations. Results from individual trials are reported in Supplementary Figs. S16 and S17 for zBMI and BMI, respectively. All trials that provided data by place of residence were from high income countries, so we were not able to perform a subset analysis on this factor.

No trials provided results subgrouped by place of residence for the older age group (12–18 years).

#### Religion

In the younger age group (5–11 years), we included two trials (767 participants) with both zBMI and BMI data subgrouped by religion. The test for interaction provided no evidence of a differential effect of interventions on zBMI (P = 0.2, Fig. 2) or BMI (P = 0.2, Fig. 3) between participants in the dominant religion group and participants in minority religion group. Results from individual trials are reported in Supplementary Figs. S18 and S19 for zBMI and BMI, respectively. Both trials that provided data by religion were from high income countries, so we were not able to perform a subset analysis on this factor.

No trials provided results subgrouped by religion in the older age group (12–18 years).

#### Occupation (parental)

In the younger age group (5−11 years), we included one trial (740 participants) with zBMI and four trials (2503 participants) with BMI data, subgrouped by parental occupation. There was no evidence of a differential effect of interventions on zBMI (P = 0.7, Fig. 2) or BMI (P = 0.9, Fig. 3), between participants in the higher occupation group and participants in the lower occupation group. Results from individual trials are reported in Supplementary Figs. S20 and S21 for zBMI and BMI, respectively. All trials providing data by parental occupation were from high income countries, so we were not able to perform a subset analysis on this factor.

No trials provided results subgrouped by parental occupation in the older age group (12–18 years).

#### Social capital

In the younger age group (5–11 years), we included only a single trial (782 participants) subgrouped by social capital. In this trial, children were divided into a subgroup in which their parents had a spouse in the same house and a subgroup with a different family structure. The test for interaction provided some evidence of a differential effect of intervention on zBMI (P = 0.08, Fig. 2) and BMI (P = 0.009, Fig. 3), between participants in the higher social capital group and participants in the lower social capital group, with the intervention appearing to be more effective in participants experiencing lower social capital. Subset results from this trial are reported in Supplementary Figs. S22 and S23. This trial was conducted in a high-income country.

No trials provided results subgrouped by social capital in the older age group (12–18 years).

## Discussion

To the best of our knowledge, this is the first meta-analysis using primary data from randomized trials of interventions to prevent obesity in children and young people to assess the potential impact of these interventions on health equity. Our findings stem from re-analysis of data, mostly provided directly by the trialists, from 81 of the eligible 244 trials included in two recent Cochrane reviews.^24^^,^^25^

We found that there were no substantial differences across PROGRESS subgroups, suggesting the interventions to prevent obesity do not have significant impacts on inequalities. We did, however, observe that interventions had more beneficial impacts on zBMI in younger boys (5–11 years) than in younger girls (based on data from 45 trials). Although the magnitude of this difference was small, the consistency in direction of this finding across zBMI and BMI, in addition to the narrow 95% confidence intervals (CIs) and small P value, supports this being a true difference. Such a difference was not observed for older children (12–18 years). This supports the findings of previous trials in primary schools of multi-component physical activity interventions.^36^^,^^37^ In the UK and many other countries, physical education at primary school is taught in mixed groups of boys and girls, while for older children there is more variation in the use of single or mixed gender physical education. Our findings suggest that interventions for younger children may benefit from being equally engaging and enjoyable for females and males. However, in the older age group, where increased BMI may correspond with decreased percentage body fat in males but not females,^38^ a more beneficial effect of interventions in males compared with females could be masked by examining BMI only. More evidence is required from studies that use better proxies of percentage body fat.

For ethnicity, the average difference in MD (based on 21 trials) between minority and dominant groups for zBMI in 5–11 years was similar in magnitude (and CIs) to the difference between boys and girls, perhaps indicating that interventions had more beneficial impacts in the dominant ethnic groups than the minority groups. However, the strength of evidence is weaker (evidenced by a larger P value, potentially due to smaller sample size) and the inconsistency with reported BMI (difference in MDs in the opposite direction) means we cannot confidently say there is a true difference from these observations. Two other signals were based on very small numbers of trials, so should be interpreted with caution. The first of these was that in the younger age group, the intervention effects on zBMI were greater for those living in a rural community compared with an urban community. One of the two trials contributing to this analysis included significant involvement from the local premier team sports (football) club, role models and family campaigns. The second was that, again in the younger age group and based on the same trial with local premier team sports club involvement, the effect of intervention on BMI was greater in children from families with low social capital.

Our results concur with previous work addressing the same question, using secondary data.^23^^,^^39^ Our findings suggest that policy makers, commissioners and providers can be confident in promoting the types of interventions to prevent obesity in children and young people included in the two Cochrane reviews that were found to have a beneficial effect on BMI or zBMI independently of PROGRESS inequity factors, thus not increasing health disparities. These reviews found beneficial effects for interventions that promoted physical activity (only) over the medium-term (9–15 months) and long-term (>15 months) (mean difference around −0.30 kg/m^2^ BMI) for adolescents aged 12–18 years and over the medium term (−0.1 kg/m^2^ BMI, −0.05 zBMI) for children aged 5–11 years. Beneficial effects were also found for interventions that promoted physical activity alongside healthy eating in the short-term (3–9 months) and medium-term (−0.11 kg/m^2^ BMI, around −0.04 zBMI) for 5–11-year-olds. Although these beneficial effects are small, when delivered at scale, the effects of these preventive interventions have shown through modelling (for England) to have the potential to contribute meaningfully to reducing the prevalence of childhood obesity.^40^

None of the included trials evaluated a ‘whole systems approach’ to prevention of obesity, despite increasing interest in such strategies.^41^ Whole systems approaches “consider the multifactorial drivers of overweight and obesity, involve transformative co-ordinated action across a broad range of disciplines and stakeholders, operate across all levels of governance and throughout the life course”.^42^ Evaluation of whole-systems approaches is possible, as exemplified by a cluster-randomized trial of the ‘Healthy Together Victoria’ initiative,^43^ which involved universal interventions complemented by targeted interventions, as suggested by Frohlich and Potvin.^18^ This trial did not meet our inclusion criteria because it did not follow up individual children for their change in BMI.

Strengths of our study include the collection of subgrouped trial data directly from the trialists when these had not been reported in articles. Our response rates of trials 58% for successful contact and 28% for data collection were much higher than we anticipated, allowing sizeable sample sizes for some of the meta-analyses. We suspect that our personalized emails and convenient data collection methods may have contributed partly to this. The studies included are comparable to the studies not included (those from which we did not manage to obtain subgroup data) with regards to risk of bias (35% versus 35% at high risk of bias, respectively), intervention type (14% versus 18% targeted diet only, respectively), age (73% versus 69% in the 5–11 age group, respectively) and country income status (84% versus 85% high income status, respectively). As perhaps expected, the studies contributing new data had a slightly more recent publication years (2005–2022) compared with studies not included (1997–2022) although the median publication year was similar (2015 versus 2014, respectively).

Requesting dichotomized for baseline inequity factors made the process of providing them simple, the meta-analyses of interactions uncomplicated (requiring minimal assumptions about the nature of the relationships across subgroups), gave us reasonable power to test our null hypotheses across as many studies as possible and led to relatively straightforward interpretation of the results as differences in mean differences. A further strength of our results is the lack of discernible heterogeneity in the interaction estimates across studies, providing some justification for the crude combination of findings from trials with very different intervention strategies.

The study is not without limitations. First, while response rates were high for this kind of research, we still included only a minority of the trials identified by the Cochrane reviews, and numbers of trials for some of the PROGRESS factors were very small indeed. Second, the simplicity of the dichotomization of inequity factors masks any complex relationships between these factors and magnitude of effect. We had originally planned to seek individual participant data from the trials, which would have allowed detailed exploration. However, this would have been a much more laborious process, potentially with a lower response rate and certainly with more administrative burden through data sharing agreements. Third, different dichotomizations were used in different trials, and most were selected by the trialists. We directly assessed the potential for bias in selection of the cut-point in our risk-of-bias assessment. Whilst we did not judge it likely that trialists had manipulated the cut-offs, it is possible that there was undetected reporting bias that may not have been there had we obtained individual participant data. Fourth, since we examined the impact of eight PROGRESS factors on two outcomes in two age groups, there is possibility of observing small P values by chance alone. To circumvent this, we have interpreted results by taking into account consistency of findings across multiple analyses as well as the size of the P value.

Finally, we planned to examine only factors related to inequity that are included in the PROGRESS framework. We did not explore other dimensions such as those represented by the wider PROGRESS-Plus framework. We knew that data on these factors, such as sexual orientation and disabilities, were unlikely to have been collected by trialists, meaning we could not investigate them reliably. We did examine dependence of intervention effects on baseline weight status and will report the results of this investigation elsewhere.

We recommend that future trialists collect PROGRESS factors (and consider collecting additional factors listed in PROGRESS-Plus) and carry out subgroup analyses to check that interventions are not increasing any health disparities that may exist. Whilst our recommendation arising from this research applies specifically to trials of interventions to prevent obesity, we believe that it extends to many other areas of health research.

## Contributors

• Conceptualization: JPT Higgins, CD Summerbell and BL Heitmann
• Formal analysis: AL Davies and JC Palmer, with lesser contributions from F Spiga and JPT Higgins (these authors accessed and verified the data)
• Funding acquisition: JPT Higgins and CD Summerbell
• Investigation: generation and contribution of trial data: all members of the *Inequity in Obesity Prevention Trialists Collaborative Group*; data collection and processing: JC Palmer and AL Davies; coding intervention characteristics: CD Summerbell; interpretation: AL Davies, JC Palmer, F Spiga, BL Heitmann, R Jago, CD Summerbell and JPT Higgins
• Methodology: JPT Higgins and AL Davies
• Project administration: JC Palmer
• Supervision: JPT Higgins
• Writing—original draft preparation: AL Davies, F Spiga, CD Summerbell and JPT Higgins
• Writing—review & editing: JC Palmer, AL Davies, F Spiga, BL Heitmann, R Jago, CD Summerbell and JPT Higgins
• All authors were responsible for the decision to submit the manuscript

## Data sharing statement

The protocol for this work was published at https://fundingawards.nihr.ac.uk/award/NIHR131572 as an appendix to the April 2023 version of the wider project protocol. Only aggregated data by subgroup were collected from the trialists. These data are presented in the figures in the Supplementary materials to the paper and are available by request from the authors.

## Declaration of interests

JCP, ALD, FS, RJ and JPTH were funded by grants from National Institute for Health and Care Research (NIHR) for this work. RJ also received funding from NIHR Applied Research Collaboration West and he is PI of a UKRI grant which does not directly relate to this work. JPTH received travel expenses funded by European Association for the Study of Obesity to give plenary talk at European Congress on Obesity in May 2023.

## Appendix ASupplementary data

Supplementary Figs. S1–S23 and Tables S1–S5

Collab author

## References

[1] Commission on Ending Childhood Obesity (2016). https://www.who.int/publications/i/item/9789241510066.

[2] N. C. D. Risk Factor Collaboration (2024). Worldwide trends in underweight and obesity from 1990 to 2022: a pooled analysis of 3663 population-representative studies with 222 million children, adolescents, and adults. Lancet.

[3] Lister N.B., Baur L.A., Felix J.F. (2023). Child and adolescent obesity. Nat Rev Dis Primers.

[4] Olsen N.J., Ostergaard J.N., Bjerregaard L.G. (2024). A literature review of evidence for primary prevention of overweight and obesity in healthy weight children and adolescents: a report produced by a working group of the Danish Council on Health and Disease Prevention. Obes Rev.

[5] UK Government Childhood obesity. POSTnote number 640, April 2021. London: the parliamentary office of science and technology. https://researchbriefings.files.parliament.uk/documents/POST-PN-0640/POST-PN-0640.pdf.

[6] Staatz C.B., Kelly Y., Lacey R.E., Hardy R. (2021). Area-level and family-level socioeconomic position and body composition trajectories: longitudinal analysis of the UK Millennium Cohort Study. Lancet Public Health.

[7] Buoncristiano M., Williams J., Simmonds P. (2021). Socioeconomic inequalities in overweight and obesity among 6- to 9-year-old children in 24 countries from the World Health Organization European region. Obes Rev.

[8] Jiwani S.S., Carrillo-Larco R.M., Hernandez-Vasquez A. (2019). The shift of obesity burden by socioeconomic status between 1998 and 2017 in Latin America and the Caribbean: a cross-sectional series study. Lancet Glob Health.

[9] Pickett K., Taylor-Richardson D. (2011). https://www.thenhsa.co.uk/app/uploads/2022/01/Child-of-the-North-Report-FINAL-1.pdf.

[10] Rafei A., Elliott M.R., Jones R.E., Riosmena F., Cunningham S.A., Mehta N.K. (2022). Obesity incidence in U.S. children and young adults: a pooled analysis. Am J Prev Med.

[11] Shah B., Tombeau Cost K., Fuller A., Birken C.S., Anderson L.N. (2020). Sex and gender differences in childhood obesity: contributing to the research agenda. BMJ Nutr Prev Health.

[12] Yeary K.H.K., Sobal J., Wethington E. (2017). Religion and body weight: a review of quantitative studies. Obes Rev.

[13] Carrillo-Alvarez E., Kawachi I., Riera-Romani J. (2019). Neighbourhood social capital and obesity: a systematic review of the literature. Obes Rev.

[14] Lorenc T., Petticrew M., Welch V., Tugwell P. (2013). What types of interventions generate inequalities? Evidence from systematic reviews. J Epidemiol Community Health.

[15] O'Neill J., Tabish H., Welch V. (2014). Applying an equity lens to interventions: using PROGRESS ensures consideration of socially stratifying factors to illuminate inequities in health. J Clin Epidemiol.

[16] Welch V., Petticrew M., Petkovic J. (2015). Extending the PRISMA statement to equity-focused systematic reviews (PRISMA-E 2012): explanation and elaboration. Int J Equity Health.

[17] McNulty M., Smith J.D., Villamar J. (2019). Implementation research methodologies for achieving scientific equity and health equity. Ethn Dis.

[18] Frohlich K.L., Potvin L. (2008). Transcending the known in public health practice: the inequality paradox: the population approach and vulnerable populations. Am J Public Health.

[19] Bronfenbrenner U. (1977). Toward an experimental ecology of human development. Am Psychol.

[20] Nuffield Council on Bioethics (2007). https://www.nuffieldbioethics.org/publications/public-health.

[21] Backholer K., Beauchamp A., Ball K. (2014). A framework for evaluating the impact of obesity prevention strategies on socioeconomic inequalities in weight. Am J Public Health.

[22] Adams J., Mytton O., White M., Monsivais P. (2016). Why are some population interventions for diet and obesity more equitable and effective than others? The role of individual agency. PLoS Med.

[23] Hillier-Brown F.C., Bambra C.L., Cairns J.M., Kasim A., Moore H.J., Summerbell C.D. (2014). A systematic review of the effectiveness of individual, community and societal level interventions at reducing socioeconomic inequalities in obesity amongst children. BMC Public Health.

[24] Spiga F., Davies AL., Tomlinson E. (2024). Interventions to prevent obesity in children aged 5 to 11 years old. Cochrane Database Syst Rev.

[25] Spiga F., Tomlinson E., Davies AL. (2024). Interventions to prevent obesity in children aged 12 to 18 years old. Cochrane Database Syst Rev.

[26] Petticrew M., Wilson P., Wright K., Song F. (2002). Quality of Cochrane reviews. Quality of Cochrane reviews is better than that of non-Cochrane reviews. BMJ.

[27] Goldkuhle M., Narayan V.M., Weigl A., Dahm P., Skoetz N. (2018). A systematic assessment of Cochrane reviews and systematic reviews published in high-impact medical journals related to cancer. BMJ Open.

[28] Oliver S., Kavanagh J., Caird J. (2008).

[29] Higgins J., Palmer J., Davies A. (2023). https://fundingawards.nihr.ac.uk/award/NIHR131572.

[30] Sterne J.A.C., Savović J., Page M.J. (2019). RoB 2: a revised tool for assessing risk of bias in randomised trials. BMJ.

[31] Deeks J.J., Higgins J.P.T., Altman D.G., Higgins J.P.T., Thomas T., Chandler J. (2019). Cochrane Handbook for systematic reviews of interventions.

[32] Higgins J.P.T., Thompson S.G., Deeks J.J., Altman D.G. (2003). Measuring inconsistency in meta-analysis. BMJ.

[33] Higgins J.P.T., Eldridge S., Li T., Higgins J.P.T., Thomas J., Chandler J. (2019). Cochrane Handbook for systematic reviews of interventions.

[34] Li T., Higgins J.P.T., Deeks J.J., Higgins J.P.T., Thomas T., Chandler J. (2019). Cochrane Handbook for systematic reviews of interventions.

[35] Cohen J. (1988).

[36] Magnusson K.T., Sigurgeirsson I., Sveinsson T., Johannsson E. (2011). Assessment of a two-year school-based physical activity intervention among 7-9-year-old children. Int J Behav Nutr Phys Act.

[37] Jago R., Sebire S.J., Davies B. (2014). Randomised feasibility trial of a teaching assistant led extracurricular physical activity intervention for 9 to 11 year olds: action 3:30. Int J Behav Nutr Phys Act.

[38] Telford R.D., Telford R.M., Welvaert M. (2019). BMI is a misleading proxy for adiposity in longitudinal studies with adolescent males: the Australian LOOK study. J Sci Med Sport.

[39] Magnee T., Burdorf A., Brug J. (2013). Equity-specific effects of 26 Dutch obesity-related lifestyle interventions. Am J Prev Med.

[40] Russell S.J., Mytton O.T., Viner R.M. (2023). Estimating the effects of preventive and weight-management interventions on the prevalence of childhood obesity in England: a modelling study. Lancet Public Health.

[41] Public Health England (2019). Guidance: whole systems approach to obesity. https://www.gov.uk/government/publications/whole-systems-approach-to-obesity.

[42] Bagnall A.M., Radley D., Jones R. (2019). Whole systems approaches to obesity and other complex public health challenges: a systematic review. BMC Public Health.

[43] Strugnell C., Orellana L., Crooks N. (2024). Healthy together Victoria and childhood obesity study: effects of a large scale, community-based cluster randomised trial of a systems thinking approach for the prevention of childhood obesity among secondary school students 2014-2016. BMC Public Health.

