# Supplementary material for: Do the effects of interventions aimed at the prevention of childhood obesity reduce inequities? A re-analysis of randomized trial data from two Cochrane reviews
Source: eClinicalMedicine. 2025 Mar 4;81:103130. doi: 10.1016/j.eclinm.2025.103130 (PMC11925530; doi:10.1016/j.eclinm.2025.103130)
Supplement: Collab author [file mmc2.docx]

## **Inequity in Obesity Prevention Trialists Collaborative Group**

Trialists: Anne Aurup , Valter Cordeiro Barbosa Filho, Mark E Benden, Lynne M. Boddy, Laura M Bogart, Blakely Brown, Angela Carlin, Diana P Pozuelo Carrascosa, Li Kheng Chai, Clare Drummy, Scott Duncan, Cara Ebbeling, Eva Martos, Stuart Fairclough, Jayne Fulkerson, Douglas A Gentile, Mary B Gruber, May Grydeland, Amy S Ha, Carla Habib Mourad, Kate Gilstad-Hayden , Douglas L Hill, Gill ten Hoor, Kiya Hurley, Alison Hurst, Nahla Hwalla, Jeannette R Ickovics, Kate Jolly, Juliana Kain, Susanne Kobel, Viktoria Anna Kovacs, Susi Kriemler, Sarahmarie Kuroko, Alberto Lana, Teresa Shamah Levy, Mairena Sánchez-López, David Lubans, Brian Lynch, Kristine A Madsen, Claude Marcus, Méndez-Gómez Humarán, Carmen Morales-Ruan, Philip Morgan, Ivan Müller, Robert Newton, Analise Nicholl, Teresia O'Connor, Russell R Pate, Sebastián Peña, Lorraine B Robbins, Jardena J Puder, Thomas Robinson, Rafaela Rosário, Richard Rosenkranz, Jennifer Sacheck, Jo Salmon, Rebecca A Seguin-Fowler, Nancy E Sherwood, Hajnalka Takacs, Rachael Taylor, Haixue Wang, Haijun Wang, Robin Whittemore, Simon Wilksch, Zenong Yin, Zhixiong Zhou.

Other project team members: Katie Breheny, Deborah M Caldwell, Sarah Dawson, Yang Gao, Frances Hillier-Brown, Rebecca K Hodder, Sofus C Larsen, Theresa HM Moore, James D Nobles, Sophie M Phillips, Jelena Savović, Fanney Thorsteinsdottir, Eve Tomlinson, Luke Wolfenden.

## **Affiliations**

Department of Nutrition, Exercise and Sports, University of Copenhagen, Denmark (Aurup A); Universidade Estadual do Ceará, Brazil (Barbosa Filho VC); Texas A&M School of Public Health, Texas A&M University, TX, Usa (Benden ME); The Physical Activity Exchange, Research Institute for Sport and Exercise Sciences, Liverpool John Moores University, UK (Boddy L); RAND Corporation, Santa Monica, CA, USA (Bogart LM); School of Public and Community Health Sciences, University of Montana, Missoula, MT, USA (Brown B); Centre for Exercise Medicine, Physical Activity and Health, Sports and Exercise Sciences Research Institute, Ulster University, Belfast, UK (Carlin A); School of Human Movement and Nutrition Sciences, The University of Queensland, Brisbane, QLD, Australia (Chai LK); Southern Health and Social Care Trust, Southern College of Nursing, Craigavon Area Hospital, Portadown, UK (Drummy C); School of Sport and Recreation, Auckland University of Technology, Auckland, New Zealand (Duncan S); New Balance Foundation Obesity Prevention Center, Boston Children's Hospital, Boston, MA, USA; Department of Pediatrics, Harvard Medical School, Boston, MA, USA (Ebbeling C); University of Physical Education, Budapest, Hungary (Eva E, Takacs H); Sport and Physical Activity Department, Edge Hill University, UK (Fairclough S); Clinical and Translational Science Institute, University of Minnesota, MN, USA (Fulkerson J); Department of Psychology, Iowa State University, IA, USA (Gentile DA); Yale School of Public Health and Professor of Psychology, Yale University, CT, USA (Gilstad-Hayden K, Ickovics JR); California State Polytechnic University Humboldt, Arcata, CA, USA (Gruber MB); Department of Physical Performance, Norwegian School of Sport Sciences, Oslo, Norway (Grydeland M); Department of Sports Science and Physical Education, The Chinese University of Hong Kong, China (Ha AS); Department of Nutrition and Food Sciences, Faculty of Agricultural and Food Sciences, American University of Beirut, Riad El-Solh, Beirut, Lebanon (Habib Mourad C, Hwalla N); The Children’s Hospital of Philadelphia, PA, USA (Hill DL); School of Geography, Earth and Environmental Sciences, University of Birmingham, Uk (Hurley K); University of Exeter Medical School, Exeter, UK (Hurst A); Department of Applied Health Sciences, University of Birmingham, UK (Jolly K); Instituto de Nutrición y Tecnología de Alimentos (INTA), University of Chile (Kain J); Ulm University Hospital, Division of Sports- and Rehabilitation Medicine, Ulm, Germany (Kobel S); Division of Nutrition Physiology and Epidemiology, National Institute of Pharmacy and Nutrition, Budapest, Hungary (Kovacs VA); Epidemiology, Biostatistics and Prevention Institute, University of Zurich, Switzerland (Kriemler S); Department of Women's and Children's Health, University of Otago, New Zealand (Kuroko S); Department of Preventive Medicine and Public Health. University of Oviedo/ISPA, Spain (Lana A); Centro de Investigación en Evaluación y Encuestas, Instituto Nacional de Salud Pública, Cuernavaca, Morelos, México (Levy TS, Morales-Ruan C); Centre for Active Living and Learning, College of Human and Social Futures, University of Newcastle, Newcastle, NSW, Australia; Faculty of Sport and Health Sciences, University of Jyväskylä, Jyväskylä, Finland; Hunter Medical Research institute, New Lambton Heights, NSW, Australia (Lubans D); Mayo Clinic Rochester, Rochester, MN, USA (Lynch B); UC Berkeley, School of Public Health, Berkeley, CA, USA (Madsen KA); Department of Clinical Science, Intervention and Technology, Karolinska Institutet, Sweden (Marcus C); Centro de Investigación en Matemáticas A.C., Unidad Aguascalientes, Aguascalientes, México (Méndez-Gómez H); Centre for Active Living and Learning, College of Human and Social Futures, School of Education, University of Newcastle, Callaghan, NSW, Australia (Morgan P); Department of Sport, Exercise and Health, University of Basel, Switzerland (Müller I); Pennington Biomedical Research Centre, Baton Rouge, LA, USA (Newton R); Exercise Medicine Research Institute, School of Medicine and Health Sciences, Edith Cowan University, Australia (Nicholl A); USDA/ARS Children's Nutrition Research Center, Huston, TX, USA (O'Connor T); Department of Exercise Science, University of South Carolina, Columbia, SC, USA (Pate RR); Finnish Institute for Health and Welfare, Mannerheimintie 166 Helsinki, Finland (Peña S); MSU College of Nursing, Michigan State University, MI, USA (Pfeiffer LB); University of Castilla-La Mancha, Faculty of Nursing, Cuenca, Spain (Pozuelo Carrascosa DP); Obstetric service, Department Woman-Mother-Child, Lausanne University Hospital, Lausanne, Switzerland (Puder JJ); Department of Pediatrics, Stanford University, USA (Robinson T); School of Nursing, Health Sciences Research Unit: Nursing (UICISA: E), Nursing School of Coimbra (ESEnfC), University of Minho, School of Nursing, Braga, Portugal. (Rosário R); Kinesiology and Nutrition Sciences, University of Nevada, Las Vegas, NE, USA (Rosenkranz R); Department of Exercise and Nutrition Sciences, Milken Institute School of Public Health, The George Washington University, DC, USA (Sacheck J); Institute for Physical Activity and Nutrition, Deakin University, VIC, Australia (Salmon J); Universidad de Castilla-La Mancha, School of Education, Ciudad Real, Spain and Health and Social Research Center, Cuenca, Spain (Sánchez-López M); Institute for Advancing Health through Agriculture, Texas A&M University System, College Station, TX, USA (Seguin-Fowler RA); Division of Epidemiology and Community Health, School of Public Health, University of Minnesota, MN, USA (Sherwood NE); Department of Medicine, University of Otago, New Zealand (Taylor R); Dept. Work and Social Psychology, Faculty of Psychology and Neuroscience, Maastricht University, The Netherlands (Ten Hoor G); Department of Maternal and Child Health, School of Public Health, Peking University, China (Wang H, Wang H); Yale School of Nursing, Yale University, CT, USA (Whittemore R); College of Education, Psychology & Social Work, Flinders University, South Australia, Australia, Advanced Psychology Services, Adelaide, SA, Australia (Wilksch S); Institute for Sport Performance and Health Promotion, Capital University of Physical Education and Sports, Beijing, China (Yin Z, Zhou Z); Population Health Sciences, Bristol Medical School, University of Bristol, Bristol, UK (Breheny K, Caldwell DM, Dawson S, Moore THM, Savović J, Tomlinson E); Department of Sport, Physical Education and Health, Hong Kong Baptist University, Kowloon, Hong Kong (Gao Y); Human Nutrition Research Centre and Population Health Sciences Institute, University of Newcastle, Newcastle, UK (Hillier-Brown F); School of Medicine and Public Health, The University of Newcastle, Australia (Hodder RK, Wolfenden G); Faculty of Medical and Health Sciences, University of Copenhagen, Copenhagen, Denmark (Larsen S, Thorsteinsdottir F); Health, Nutrition & Environment, Leeds Beckett University, UK (Nobles JD), Department of Sport and Exercise Science, Durham University, Durham, UK and Child Health and Physical Activity Laboratory, School of Occupational Therapy, Western University, London, Ontario, Canada (Phillips SM).
